# Self-regulation of frontal-midline theta facilitates memory updating and mental set shifting

**DOI:** 10.3389/fnbeh.2014.00420

**Published:** 2014-12-05

**Authors:** Stefanie Enriquez-Geppert, René J. Huster, Christian Figge, Christoph S. Herrmann

**Affiliations:** ^1^Experimental Psychology Laboratory, Department of Psychology, European Medical School, Carl von Ossietzky University of OldenburgOldenburg, Germany; ^2^Karl-Jaspers Clinic, European Medical School Oldenburg-GroningenOldenburg, Germany; ^3^Research Center Neurosensory Science, Carl von Ossietzky UniversityOldenburg, Germany; ^4^Center for Excellence ‘Hearing4all’, Carl von Ossietzky University of OldenburgOldenburg, Germany

**Keywords:** frontal-midline theta, neurofeedback, cognitive enhancement, executive functions, EEG, proactive and reactive control

## Abstract

Frontal-midline (fm) theta oscillations as measured via the electroencephalogram (EEG) have been suggested as neural “working language” of executive functioning. Their power has been shown to increase when cognitive processing or task performance is enhanced. Thus, the question arises whether learning to increase fm-theta amplitudes would functionally impact the behavioral performance in tasks probing executive functions (EFs). Here, the effects of neurofeedback (NF), a learning method to self-up-regulate fm-theta over fm electrodes, on the four most representative EFs, memory updating, set shifting, conflict monitoring, and motor inhibition are presented. Before beginning and after completing an individualized, eight-session gap-spaced NF intervention, the three-back, letter/number task-switching, Stroop, and stop-signal tasks were tested while measuring the EEG. Self-determined up-regulation of fm-theta and its putative role for executive functioning were compared to an active control group, the so-called pseudo-neurofeedback group. Task-related fm-theta activity after training differed significantly between groups. More importantly, though, after NF significantly enhanced behavioral performance was observed. The training group showed higher accuracy scores in the three-back task and reduced mixing and shifting costs in letter/number task-switching. However, this specific protocol type did not affect performance in tasks probing conflict monitoring and motor inhibition. Thus, our results suggest a modulation of proactive but not reactive mechanisms of cognitive control. Furthermore, task-related EEG changes show a distinct pattern for fm-theta after training between the NF and the pseudo-neurofeedback group, which indicates that NF training indeed tackles EFs-networks. In sum, the modulation of fm-theta via NF may serve as potent treatment approach for executive dysfunctions.

## Introduction

Time-frequency analyses of electroencephalographic (EEG) recordings reveal synchronous processes of neural networks known as neuronal oscillations. Nowadays, these neural oscillations are considered to provide a linkage of neural activity with behavior and thought. As such, they are supposed to coordinate neuronal spiking between and within brain circuits (e.g., Buzsáki, 2006; Buzsáki et al., 2013), whereby different neural oscillations may appear at the same time and interact with each other in a hierarchical way in order to implement perception and cognition (Herrmann et al., 2004; Canolty et al., 2006; Basar and Güntekin, 2008; Fingelkurts and Fingelkurts, 2014).

Regarding higher cognitive functions, frontal-midline (fm) theta oscillations are of particular interest. Fm-theta oscillations are recorded over fronto-medial brain regions at frequencies between 4–8 Hz and are suggested to be generated in the midcingulate cortex (MCC; Mitchell et al., 2008; Cavanagh and Frank, 2014), a highly interconnected brain structure (Beckmann et al., 2009; Vogt, 2009) that is part of the superordinate cognitive control network (Niendam et al., 2012). The MCC is known to be crucially involved in executive functioning (Cavanagh et al., 2012), which enables goal directed behavior (e.g., Lezak, 2012). Increases of fm-theta power have been associated with enhanced coupling between neuronal spikes and the phase of the population theta cycle, and thus are suggested to organize neural processes during decision points where executive functioning is needed and information is integrated to inform action selection (Cavanagh and Frank, 2014). Enhanced cognitive processing is accompanied with increases of fm-theta (Mitchell et al., 2008), specifically in tasks involving working memory (WM; Mitchell et al., 2008) and executive functions (EFs; Nigbur et al., 2011). In addition, fm-theta activity has been related to efficient WM maintenance (Tóth et al., 2014), and increases of fm-theta activity during task processing have been shown to predict successful behavioral performance (Sederberg et al., 2003) and RTs in a Simon task involving conflict monitoring (Cohen and Donner, 2013). Correspondingly, the absence of fm-theta up-regulation, when executive functioning is required, has been reported to be associated with reduced performance (e.g., Donkers et al., 2011). In short, fm-theta has been proposed as a universal mechanism for EFs with the MCC acting as hub for the integration of relevant information (Cavanagh et al., 2012).

The possibility to modulate neural oscillations marks an important step beyond simply focusing on correlations between oscillations and cognitive performance. The feasibility to self-regulate endogenous neural activity has been nicely demonstrated in extracellular recordings for activity of single neurons (e.g., Olds and Olds, 1961), local field potentials (LFPs) in the animal model (e.g., Sterman et al., 1970), as well as for EEG activity measured on the scalp of humans (Kamiya, 1968). Nowadays the technique by which the modulation of neuronal activity is achieved in a reward-based fashion by giving feedback on the real-time status of the participants’ brain activity who thereby learn to voluntarily control it, is termed neurofeedback (NF; Sherlin et al., 2011; Huster et al., 2014). NF-systems belong to a subclass of brain computer interfaces (BCIs) that aim at the regulation of oscillations and slow cortical potentials with EEG (Birbaumer et al., 2009) and magnetoencephalography (MEG; e.g., Sudre et al., 2011), or at the modulation of brain metabolism by functional magnetic resonance imaging (fMRI; e.g., Birbaumer et al., 2013; Ruiz et al., 2013) and near infrared spectroscopy (NIRS; e.g., Mihara et al., 2012; Kober et al., 2014). The modulation of neural oscillations by EEG-NF has been shown for diverse frequencies in association to different cognitive (sub) processes (see review of Gruzelier, 2014), for instance regarding enhanced upper alpha band and improved mental rotation (e.g., Hanslmayr et al., 2005; Zoefel et al., 2011), increased gamma-band activity and enhanced episodic retrieval (Keizer et al., 2010), or enhanced sensorimotor rhythm (SMR) and improved declarative learning (Hoedlmoser et al., 2008).

With respect to fm-theta oscillations, its basic modulability by NF has recently been demonstrated (e.g., Enriquez-Geppert et al., 2014). van Schie et al. (2014) designed a study to investigate effects of controlled fm-theta down-and up-regulation on WM performance. They demonstrated that the up-regulation led to increased, the down-regulation to attenuated performance. Concerning fm-theta NF training, positive effects on cognition have been shown for WM and attention (Wang and Hsieh, 2013). Notwithstanding this, effects of fm-theta modulation on EFs by NF remain to be elucidated.

The modulation of fm-theta by NF can be categorized within a broader framework of theta protocols. Generally, theta protocols can be performed at diverse electrode positions thereby focusing different neural networks (see review Gruzelier, 2014). A subdivision of protocols pertains to NF for theta up- or theta-downregulation. In case of the clinical application, down regulation of theta is most often combined with the modulation of other frequencies (e.g., theta-beta training, e.g., Gani et al., 2008; Arns et al., 2013), as for example seen with attention deficit hyperactivity disorder (ADHD). Here, EEG deviations have been observed during resting state compared to healthy controls, namely increased theta activity in context of beta activity (e.g., Chabot and Serfontein, 1996; Clarke et al., 2001). However, this deviation of theta measured at rest reflects a tonic condition that has to be dissociated from phasic theta responses, which in turn have been linked to specific cognitive functions. An important fact concerning the dissociation of tonic and phasic amplitude is the notion that successful behavior seems to be related to both of these EEG phenomena as good performance is related to a decrease in tonic and increased phasic theta activity (see review, Klimesch, 1999).

Taken together, NF seems perfectly suited to induce positive effects on EFs. This issue becomes imperative since EFs are crucial for success in daily life as they mediate learning processes (St Clair-Thompson et al., 2010), the control of emotions (e.g., Fikke et al., 2011), and predict academic achievement and social functioning (e.g., Miller et al., 2012b). Furthermore, age-related declines in EFs have been reported to lead to reduced success in everyday activities (Vaughan and Giovanello, 2010). Moreover, disturbances of EFs are associated with neurocognitive, and psychiatric impairments (e.g., Elliott, 2003).

The aim of this study is therefore to investigate the behavioral and neuronal effects of fm-theta NF on EFs. We set up an eight session personalized NF training for up-regulation of fm-theta and compared the achieved training effects to those of a pseudo NF training constituting an active control condition. A pre/post-test training design was implemented measuring the four most independent and representative EFs (Miyake et al., 2000; Miyake and Friedman, 2012) namely memory updating, set shifting, conflict monitoring, and motor inhibition, experimentally operationalized by a three-back, a number-letter task-switching, a Stroop-, and a stop-signal task. Concurrently, the participants’ EEG was recorded. We hypothesized that NF for self-up-regulation of fm-theta would lead to enhanced performance in EFs at the behavioral level, and would also translate to enhanced fm-theta during executive functioning in the respective tasks compared to the pseudo NF group.

## Material and methods

### Participants

40 students (19 men; mean age: 24.8, standard deviation: 3.3) participated in this study. Participants were pseudo-randomly assigned to the experimental (NF; *n* = 19, 8 men; mean age: 23.8, standard deviation: 2.7) or the active control group (pseudo-NF, *n* = 21, 11 men; mean age: 25.8, standard deviation: 3.8) in order to balance the groups according to age, education level and gender. All subjects were right handed, as indicated by the Edinburgh Handedness Inventory (Oldfield, 1971), and had normal or corrected to normal vision. None of them reported a history of psychiatric or neurological disorders. Before the experiment, all participants gave written consent to the protocol approved by the ethics committee of the University of Oldenburg. For study participation, participants were rewarded with 8 € per hour. The study was conducted in accordance with the Declaration of Helsinki.

### Pre-post training design

To measure the effects of up-regulation of fm-theta on EFs, an individualized and adaptive eight session NF training was performed on consecutive working days within 2 weeks, comparing the effects to an active control group, the so-called pseudo-NF. One day before and one day after finishing the NF training, an EFs test-battery, assessing the four most important and independent EFs. The study was carried out at the Carl von Ossietzky University of Oldenburg.

#### Executive function test-battery, EEG recordings and preprocessing

**Figure 1:**
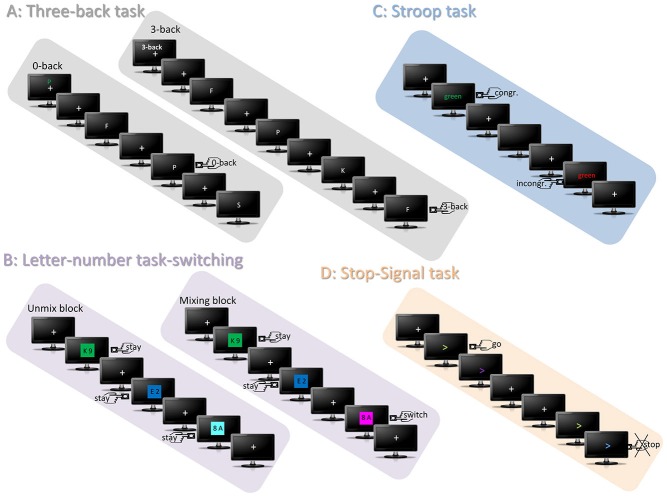
**Depicts all four tasks of the EFs test-battery**. The graphic depicts the targets of each task by a left/right button-press icon. **(A)** Represents the trial sequence of the three-back tasks that consisted of the alternating presentation of the zero-back and the three-back sequence. Each sequence started with a fixation cross, above which either a green letter indicated the subjects to perform the zero-back task and to detect in the following sequence targets that matched to the green letter or above which “3-back” indicated to update every letter and to compare it to the identity of three trials before. **(B)** Represents the letter-number task switching. In the unmix-block, the colors green, blue and turquoise indicate that only one categorization task has to be performed, for instance only number categorization. In the example “unmix-block” depicted in the graphic, uneven numbers require a right hand button-press and even a left hand button-press. In the mixing-block the colors of the other category are also introduced and indicate that categorization has to be performed concerning both letters and numbers, each respectively. Thus, the third trial of the depicted example “mixing-block” requires a “switch” of number categorization to letter categorization and thereby leads to another required response, since vocals have to be responded with the right hand. **(C)** Represents the trials of the Stroop task requiring the categorization of the presentation color (e.g., right button press for presentation colors), but not the word-meaning. Consequently, although the word meaning in this example is the same, the required button-press is different because of the different presentation color of the word. **(D)** Shows exemplarily two trials of the visual stop-signal task, whereby the directions of the arrows are indicating the response side. In this example the color change into purple should be ignored, however, the change into blue indicates to stop the initiated response.

In the first task of the EFs test-battery (see Figure 1), the visual three-back task, participants were presented with two kinds of letter sequences, the three-back and the zero-back sequence. In the three-back sequence, participants were instructed to respond via button press (using the right index finger), whenever a letter had already been presented three trials before the current one (three-back target condition). In the zero-back condition subjects were instructed to simply respond whenever a letter matched a target letter presented at the beginning of the letter sequence. In all other cases, participants were asked not to react. There were ten three-back and nine zero-back sequences that were presented in an alternating fashion with 24 white letters presented on a black background for 1000 ms, and followed by a fixation cross with a duration of 1000 ms (total trial length: 2000 ms; total trial number per sequence: 24; target numbers per sequence: 8). It is assumed that the three-back condition requires memory updating processes whereas the zero-back condition does not.

The number-letter task-switching consisted of number-letter pairs that were presented on a colored background. Participants were instructed to either classify the numbers (in even or odd numbers) or the letters (in vowels or consonants) by a right or left index button press. The classification depended of the specific background color (red, orange, pink vs. green, blue, and turquoise). Two versions with different number-color and letter-color assignments were utilized. Letter-number pairs were presented for 2000 ms followed by a white fixation cross presented for 1000 ms (trial length: 3000 ms). The task consisted of two parts. In the first part that included unmix-blocks, only the letters (unmix-block 1; 60 trials) had to be classified, followed by a second block during which only numbers (unmix-block 2; 60 trials) had to be processed. In the second part that included mixing blocks, participants had to switch between both classification types (trial number: 234; switches: 70). In contrast to the unmix-blocks, the mixing blocks required the switching between the two categorization tasks, thus it involves set-shifting.

The Stroop task contained the presentation of the color-words blue, green, yellow, and red, which were either presented congruently in colors matching the word meaning (e.g., the color-word “blue” presented in blue) or incongruently in an un-matching fashion (e.g., the color-word “blue” presented in red). Participants were instructed to indicate via a right or left index button press the presentation color, but not the word-color. A trial started with a fixation cross presented for a randomized duration of 1200–1400 ms, and followed by the color word for 500 ms. Afterwards a second fixation cross was presented for a randomized duration of 100–500 ms. Trials were separated by an inter-trial interval (ITI) of a randomized duration of 400–800 ms (total trial number: 128, number of incongruent trials: 64). Regarding the involved EFs, conflict-monitoring is supposed to detect the conflict associated with incongruent trials relative to the congruent condition.

In the visual stop-signal task, left- or rightward pointing arrows were presented that changed their color during presentation time from purple to green, blue, or orange. Participants were instructed to press the button of a two-button box according to the arrow direction either with the right or the left index in a fast and accurate way directly after beginning of the arrow presentation (go-trials). However, a specific color change (for instance from purple to orange) was indicating that participants had to abort their initiated response (stop-trials). Two versions with different color-response assignments were used. The timing of the color-change was adjusted dynamically via a certain stimulus onset asynchrony (SOA; Logan et al., 1997) such that participants could stop their response in 75% of the stop-trials. Every trial with a length of 2 s started with a fixation cross that was randomly set with a duration of 300–600 ms. Then, the arrow was presented with an initial duration of 250 ms (this SOA was adjusted by adding 50 ms after every second correct or subtracting 50 ms after every failed stop trial to reach the intended error rate) before the color changed. The color change remained on screen for another 200 ms. The trial was ended with the presentation of a fixation cross (total number of trials: 300; number of stop trials: 100). The stop-trials of this task require motor inhibition as EFs, in contrast to motor execution during go-trials.

Before each task, a short exercise period was implemented to familiarize participants with the task requirements and the presentation. The four tasks each had a duration of roughly eight to 18 min. All four EFs tasks were implemented using the Presentation software^1^.

The measurements of the EFs test-battery, as well as the NF training, were conducted in an electrically shielded and sound attenuated room. EEG recordings were done using the Brain Vision Recorder software and a Brain Amp EEG amplifier (Brain Products GmbH, Filching, Germany). Electrode impendences were kept below 5 kΩ for the continuous EEG recording, data was sampled at 500 Hz, and a low-pass online filter of 250 Hz was used. During the measurement of all four EFs, EEG activity was recorded from 32 Ag/AgCl electrodes (Easycap, Falk Minov Services, Munich, Germany), placed in accordance with the extended version of the international 10–20 system, with a nose electrode as online reference, and an electrode attached beneath the right eye for recording the electrooculogram (EOG) and quantification of ocular artifacts.

The offline-preprocessing was performed using the EEGLAB software^2^ and included the following steps. First, data were low-pass (80 Hz) and high-pass filtered (0.5 Hz), and then down-sampled to 250 Hz. To correct for ocular artifact, the Infomax ICA algorithm (Bell and Sejnowski, 1995; Makeig et al., 2004) was subsequently used on the continuous data. Blink-related independent components (ICs) were identified by comparing the IC activity to the eye blink artifacts in the EOG. The corresponding topographical maps of putative ICs had to show a frontal distribution. Eye-blink related ICs were then excluded from back-projection to the EEG channels. Afterwards, stimulus-locked epochs were computed comprising an interval of −1250 to +1250 ms corresponding to the stimulus presentation. Trials were then baseline-corrected. To correct for residual artifacts, a semi-automatic correction procedure was used based on the epoched data. Thereby single trials that crossed a self-set threshold (set to 60 μV) were marked automatically for visual inspection and rejection. Thereby 5.3 epochs were rejected on average per condition and task. Incorrect responses were discarded from further analyses.

Afterwards, so-called event-related spectral perturbations (ERSPs) were calculated for each task that represent log-transformed changes of power in dB relative to the baseline (Delorme and Makeig, 2004). For this time-frequency decomposition, a sinusoidal wavelet transform, using an increasing number of cycles with increasing frequency was used (range: 1–50 Hz; starting with 1 cycle at 1 Hz and increasing by 0.5 Hz per frequency; using 300 frequency steps). To visualize power changes relative to the pre-stimulus activity, the average power across the trials was divided by the frequency specific baseline values separately for each frequency. Mean ERSP values were calculated for the fm ROI over electrodes Fz, FC1, FC2, Cz.

#### Individualized and adaptive neurofeedback training

For up-regulating fm-theta by NF, eight 30-minutes training sessions were conducted. Because it was shown that fm-theta exhibits large inter-individual variability, but high intra-individual stability (e.g., Näpflin et al., 2008), a procedure for the detection of the individual fm-theta peak was used. Thus, the dominant individual fm-theta frequency peak was estimated based on the average of four peaks (+/−1 Hz), detected by the ERSPs computed from the four EF tasks. Fm-theta was shown to fall into the time range of the N200/P300 complex (Huster et al., 2013), as well as fm-negativities (Huster et al., 2013), and furthermore to have a maximum at Fz (Ishihara et al., 1981; Cavanagh et al., 2012). Thus, the individual fm-theta frequency was determined from electrode Fz, between 4–8 Hz in the corresponding ERP time range of conditions requiring EFs. Each of the eight NF sessions furthermore consisted of six five-minute blocks of NF with self-paced breaks in-between. Before and after these blocks, a five minute start/end-baseline was measured to assess resting state activity. Frequency spectra were computed using a fast Fourier transformation and a hamming window for data segments of 2 s, shifted along the data in steps of 200 ms. For NF, the software NF Suite 1.0 (Huster et al., 2014) was used.

In the following, all five steps of the basic set-up for NF (Huster et al., 2014), forming the real-time processing pipeline, are described. First, the data acquisition was based on five electrodes placed at positions Fz, FC1, FC2, FCz, and Cz, thus covering the fm-brain region, with the nose as reference, and Fp1 and Fp2 for monitoring ocular activity. Before the start-baseline measurement, an EOG calibration method (3 min) was implemented that calculates the subject-specific, artifact-associated frequency band. This was used for all following measurements for eye blink detection and rejection during further measurements (for details see Huster et al., 2014). The second step refers to the data online-preprocessing. A crucial feature of this step is the monitoring of artifacts, specifically that of eye activity. This technique is important to avoid falsely modulating eye- rather than actual brain activity, since the power of eye activity unfolds in several frequency bands. Thus, the subject-specific artifact-associated frequency band that was calculated in the EOG calibration measure was monitored. Whenever the mean amplitudes of a 2 s segment was higher than the subject-specific artifact-associated frequency band (minus one standard deviation), the segment was rejected and not used for feedback. Third, for feature generation and selection, the raw power value of each 2 s segment for the individualized fm-theta frequency was compared to the baseline power of the same frequency. Fourth, to feed back the extracted feature to the participant, a visual procedure was used in form of a visual display of a colored square (range: highly saturated red over gray to highly saturated blue, each with 40 color steps). The color saturation depended on the fm-theta activity changes, and was updated every 200 ms. The color red indicated an fm-theta power increase, blue a decrease, and gray either an eye blink or no power change relative to the start-baseline of the specific NF session. The feedback saturation scale enclosed 95% of the amplitude range, whereby values above 97.5% or below 2.5% were indicated by a maximum red or blue saturation. Fifth, participants were instructed to color the square as red and as often as possible. To do so, they received a list of strategies based on the NF literature and were encouraged to find own self-invented strategies.

Participants of the pseudo-NF were matched to participants of the actual NF group according to age and gender. During a NF block, they received a playback of the same session and block of their matched participant. Thereby, both groups received a similar visual stimulation. To enhance the credibility of the pseudo manipulation, participants of the pseudo-NF received real feedback of their own eyeblink activity, by haltering the feedback replay and introducing the gray color feedback as it is done in the actual NF group.

### Data analysis

#### NF training effects

To analyze the self-upregulation of the individual fm-theta (ind-fm-th) by NF, the relative change of the fm-theta amplitude was quantified for all training sessions during training blocks as change in percent relative to the values of the first training session. This procedure was chosen because it minimizes some of the inter-subject variability caused by unspecific effects such as amplitude variability due to the visual stimulation during feedback. Thus, this specific calculation has the advantage to compare fm-theta increases relative to a condition in which subjects are also up-regulating their brain activity, but with lower success, thereby making sure that the same system or process is engaged. However, as a drawback, it attenuates some of the relevant inter-group variance (by essentially removing training effects of the first session), therefore reducing the likelihood to find a group by session interaction. Correspondingly, a main effect is expected, as the dependent variable represents a difference measure, indicating that proper NF induces increased fm-theta not seen with the pseudo-NF training. Consequently, NF training effects were investigated by a repeated-measures ANOVA with the factors SESSION (2–8) and GROUP (NF vs. pseudo-NF group). These calculations and analyzes were also performed for the alpha (ind-fm-th + 2 Hz to ind-fm-th + 7 Hz) and beta (ind-fm-th + 7 Hz to ind-fm-th + 15 Hz) frequency bands. In case of violations of sphericity, Greenhouse-Geisser corrections were performed, and corrected *p*-values and *ε*-values are reported.

#### Data analysis: behavioral performance of executive function tasks

To investigate transfer effects of NF training on cognitive performance, mean RT and mean accuracy of correct responses were calculated for all conditions of the four EFs tasks, namely for the three-back vs. zero-back conditions of the three-back task; the unmix vs. stay vs. switch conditions of the letter number task-switching task, the congruent vs. incongruent conditions of the Stroop-task, and the go vs. stop conditions of the stop-signal task. Extreme values exceeding the mean by more than 2.5 standard deviations were excluded for statistical analyses. To investigate performance gains after NF training, performance differences between pre- and post measurements were calculated for all the conditions of the four EF tasks. A stronger performance gain was expected in conditions requiring EFs. In other words, concerning the three-back task, performance gains are expected after NF in the three-back condition, because for this condition a higher load of memory-updating is involved compared to the zero-back condition (larger effects in NF compared to pseudo-NF training). Similarly, larger effects were expected in the stay and switch conditions compared to the unmix condition after NF-training relative to the pseudo-NF training. Likewise, stronger performance gains regarding conflict monitoring were expected after NF in the incongruent compared to the congruent condition of the Stroop task. Finally, behavioral performance gains were expected in the stop condition as reflected in reduced SSRT after proper NF. Thus, pre-post measurement differences were tested for by means of independent-samples *t*-tests comparing the training and the control group. To examine the range of significant results, Cohen’s d (Cohen, 1988) was calculated for the pretest-posttest differences or the standardized mean difference in performance between pre- and post measurements (the difference was divided by the pooled standard deviation for the measurements occasions) for each group and corrected for small sample bias using the Hedges and Olkin (Hedges and Olkin, 1985) correction factor (d’) only for significant results. As result, a pre-post measurement effect size (ES) of *d*’ = 1 reflects a mean difference between pre-and post-measurements of one standard deviation.

#### Data analysis: effects of NF training on fm-theta in executive functions tasks

For the evaluation of NF effects on fm-theta during the four EFs tasks, ERSP values were extracted for the time range used for the detection of the individual fm-theta peaks (three-back task: 100–300 ms, number/letter task-switching: 100–300 ms, Stroop: 220–500 ms, stop-signal task: 300–500 ms) and the theta frequency range of 4–8 Hz. These values were then averaged for each condition, task and subject. Then, difference scores were calculated by subtracting the values before from values after training: (pre-post difference of letter number task-switching, the congruent and incongruent pre-post-differences of the Stroop-task, and the go and stop pre-post-differences of the stop-signal task. To investigate if these difference scores differed significantly between the NF and the pseudo-NF group, a MANOVA with the above-mentioned pre-post-difference scores as dependent variables and GROUP (NF vs. pseudo-NF group) as independent variable was run.

## Results

### NF training effects

**Figure 2:**
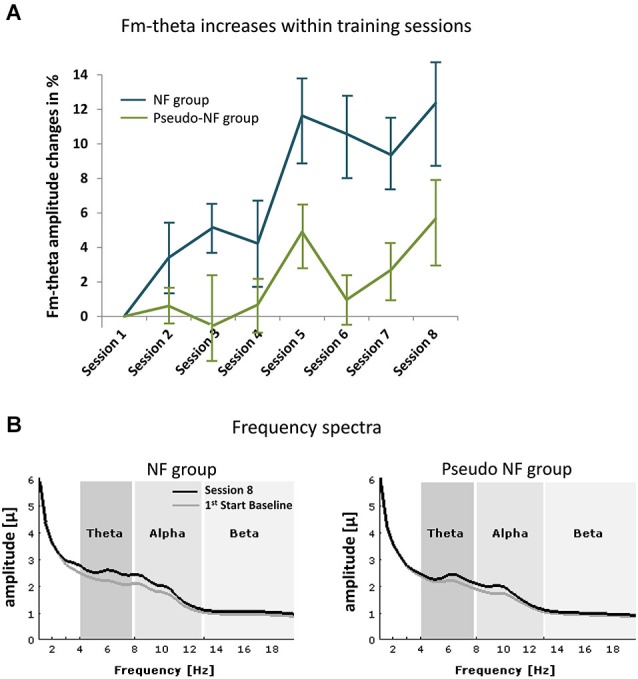
**shows frequency effects during NF. (A)** Depicts the means of fm-theta increases of the NF (blue lines) and the pseudo-NF group (green) within the eight training sessions (error bars represent standard error of mean). In **(B)** the frequency spectra are shown for the start baseline measure of the first session (in gray) and during the last training session (S8, black) for each group (left side: NF, right side: pseudo-NF group). Unspecific alpha effects are observed in both groups, whereas fm-theta is specifically increased in the experimental group.

NF training effects on the fm-theta amplitude are depicted in Figure 2. The repeated-measures ANOVA resulted in a main effect of SESSION (*F*_(4.346,39)_ = 6.487, *ε* = 0.724, *p* < 0.001) showing that every training session let to a change in fm-theta self-upregulation. The further expected main effect was the GROUP (*F*_(6,39)_ = 6.225, *p* < 0.05) effect, demonstrating the validity of the experimental manipulation resulting in stronger enhancements of activity in the NF group regarding fm-theta amplitude, starting already at the second training session when compared to the pseudo-NF group.

Regarding the alpha range, a trend for a main effect of SESSION (*F*_(6,37)_ = 8.173, *p* < 0.1) was observed, no further effects were detected. Similarly, within the beta frequency range also only a trend for the main effect of SESSION (*F*_(6,37)_ = 3.157, *ε* = 0.308, *p* < 0.1) was observed.

### Behavioral performance in executive functions tasks

**Figure 3:**
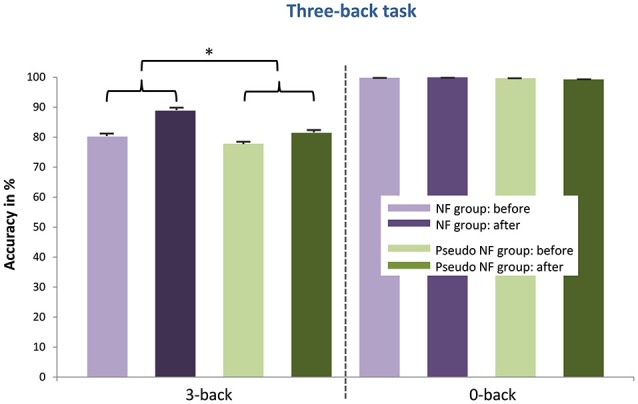
**Provides the mean accuracy scores (and standard error of mean) in the three-back task of the NF group (left) and the pseudo-NF group (right) for each condition (three-back vs. zero-back) before and after the training intervention**. Significant differences (marked with a star) between accuracy enhancements (pre- post differences as depicted with curly brackets) were detected in the three-back condition between both groups showing stronger improvements after proper NF training.

**Figure 4:**
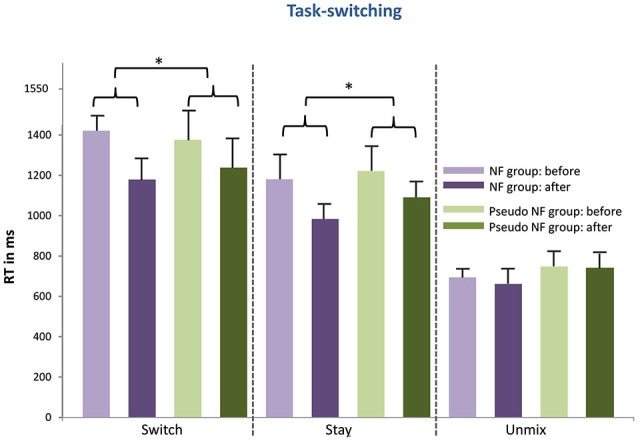
**Shows the behavioral results of the task-switching (mean RT and standard error of mean) for the NF group (left) and the pseudo-NF group (right) for all three conditions (unmix, stay, switch) before and after the training intervention**. Significant differences (pre-post differences as depicted with curly brackets) are marked with stars and are depicted between both groups as training results. Stronger enhanced RTs can be observed for the NF group in the stay and switch condition.

**Figure 5:**
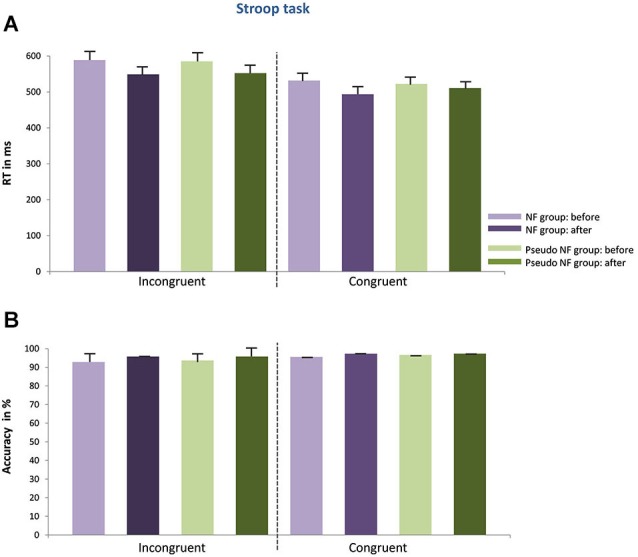
**Depicts the behavioral results of the Stroop task (mean RT in (A), accuracy scores in (B) including standard error of mean) for the NF group (left) and the pseudo-NF group (right) for both conditions (congruent, incongruent) before and after the training intervention**. No behavioral differences between groups can be observed as a result of the training intervention neither concerning RTs nor accuracy scores.

**Figure 6:**
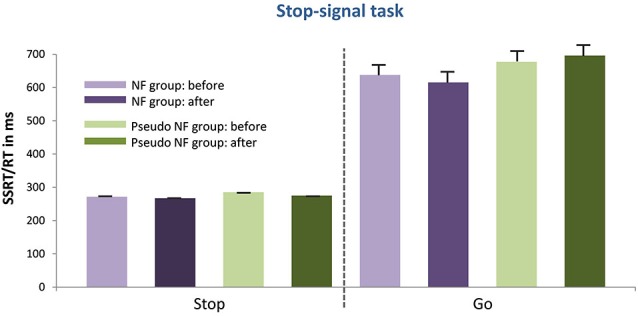
**Presents the mean RTs or the SSRT (as well as the standard error of mean) of the stop signal task for the NF group (left) and the pseudo-NF group (right) for the go and stop condition before and after the training intervention**. No performance differences between groups due to the intervention were shown.

The results of the pre- and post measurements (RTs and accuracy) are shown in Figures 3–6 computed from correct responses of all conditions for the three-back, task-switch, Stroop-, and stop-signal tasks for both training types (NF and pseudo-NF). Descriptively, these figures hint to behavioral changes, particularly in the three-back and task-switching tasks. Although these enhancements are more pronounced in the NF group, behavioral changes appear also in the pseudo-NF group. However, any study that is using a pre-post design has to expect repetition-related effects that may simply arise from repeatedly testing a task. Such unspecific repetition-related effects are particularly well known in behavioral training studies and are found in all subjects, for instance in task-switching (e.g., Karbach and Kray, 2009) or three-back tasks (e.g., Schneiders et al., 2011). Therefore, the utilization of a control group is urgently needed to dissociate such repetition-related or non-specific effects from true enhancements of cognitive functions, which in turn are reflected in stronger improvements in the intervention group than in the control group (Campbell and Stanley, 1963; Oken et al., 2008).

Concerning the three-back task, the expected stronger increases in accuracy was statistically confirmed in the three-back condition (*t*_(38)_ = 1.786; *p* < 0.05) after proper NF training when compared to the pseudo-NF training; such effects were absent in the zero-back condition (see Figure 3). These results were supported by the pretest-posttest ES. ES were larger after NF training (three-back: *d*’ = 1.059) than after pseudo-NF training (three-back: *d*’ = 0.346). For the zero-back condition, the following ES are reported (NF training: *d*’ = −0.076; pseudo-NF training: *d*’ = −0.494).

I. As expected, stronger RT decreases were observed in the switch condition (*t*_(38)_ = 1.966; *p* < 0.05), and in the stay condition (*t*_(38)_ = 1.695; *p* < 0.05) after proper NF training as compared to the pseudo intervention (see Figure 4). No differences were found for neither the unmix condition (*t*_(38)_ = 1.036; *p* = n.s.) between both training types nor for accuracy scores (switch: *t*_(38)_ = 1.633; *p* = n.s.; stay *t*_(38)_ = −1.651; *p* = n.s. unmix: *t*_(38)_ = −0.388; *p* = n.s.). These results were supported by the pretest-posttest ES. For both, switch and stay conditions, EF were larger after NF training (switch: *d*’ = 1.67; stay: d’ = 1.09) than after pseudo-NF training (switch: *d*’ = 0.582; stay: *d*’ = 0.649). However, no differences in performance gains were shown in accuracy (switch: *t*_(38)_ = 1.633, *p* = n.s.; stay: *t*_(38)_ = −1.651, *p* = n.s.). ES for the unmix condition were the following, NF training: *d*’ = −0.408 and pseudo-NF training: *d*’ = −0.063, (the ES for differences in accuracy scores are also given subsequently, NF training: switch *d*’ = 0.607, stay *d*’ = 0.718, unmix *d*’ = 0.091; pseudo-NF training: switch *d*’ = 0.879, stay *d*’ = 0.929 unmix *d*’ = 0.142 ).

II. Different from what was expected, no differences were observed when comparing NF to pseudo-NF training regarding the Stroop task, neither concerning RTs (incongruent: *t*_(34)_ = 0.247, *p* = n.s.; congruent: *t*_(38)_ = 0.142, *p* = n.s.) nor accuracy (incongruent: *t*_(38)_ = 0.989, *p* = n.s.; incongruent: *t*_(38)_ = 0.81, *p* = n.s.), see Figure 5. Similarly, low ES values were recorded for the NF and the pseudo-NF training (RTs differences in the incongruent condition: NF training: *d*’ = 0.337 and pseudo-NF training: *d*’ = 0.749; RTs differences in the congruent condition: NF training: *d*’ = −0.469 and pseudo-NF training: *d*’ = −0.143; accuracy differences in the incongruent condition: NF training *d*’ = 0.846 and pseudo-NF training *d*’ = 0.55; accuracy differences in the congruent condition: NF training: *d*’ = 0.42 and pseudo-NF training: *d*’ = 0.244).

III. Similarly, in the stop-signal (see Figure 6) task no differences were observed comparing the pre-post difference of the NF- and the pseudo-NF-training groups neither concerning SSRTs of the stop condition (*t*_(38)_ = −1.558, *p* = n.s.) nor RTs of the go-condition (*t*_(36)_ = 1.65, *p* = n.s.). Accordingly, low ES scores were registered: differences in the stop condition: NF training *d*’ = 0.159, pseudo-NF training *d*’ = 1.147; differences in the go condition: NF training *d*’ = 0.159, pseudo-NF training *d*’ = 0.147.

### Effects of NF on fm-theta in executive function tasks

In the following, a descriptive overview will be given for each task and group before and after the training; thereafter the fm-theta effects will be reported, as computed within the multivariate analysis framework.

**Figure 7:**
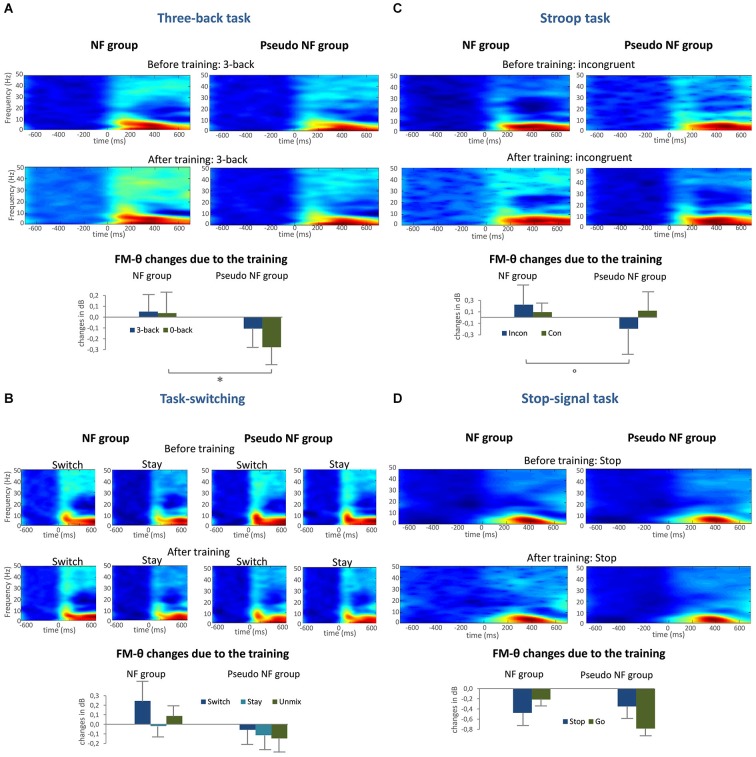
**Provides the mean ERSPs before and after training, as well as the extracted fm-theta changes (and standard error of mean) for all conditions as bar charts for the three-back condition in the three-back task (A), the switch and stay condition of the task-switching (B), the incongruent condition of the Stroop task (C), and the stop condition of the stop-signal task (D), significant univariate results are marked with a star, trends with a circle**.

I. Irrespective of group, power enhancements can especially be observed in the delta and theta frequency range around 300 ms post stimulus onset, accompanied by power reductions in the beta and slight power enhancements in the gamma range in the three-back condition of the three-back task (see Figure 7), which are in agreement with those obtained by e.g., Pesonen et al. (2007). Slight frequency changes can be observed in both groups after training.

II. The ERSPs in the task-switching task before and after the training intervention are characterized by a distinct theta-alpha power enhancement at around 200 ms, accompanied by weaker effects with a similar time pattern in the beta and gamma frequency range with a duration of approximately 100 ms, in accordance with previous studies as Prada et al. (2014). In the subsequent time frame, the power enhancement of theta and delta remains stable and the beta power reduction (20 Hz) becomes apparent.

III. To turn to the ERSPs in the Stroop task, here theta and beta power enhancement effects are observed corresponding with prior finding in literature (e.g., Cavanagh et al., 2012) beginning at 180 ms, whereby the theta power enhancement becomes maximal at 300 ms and remains stable until the end of the analyses time window. Minor frequency changes can visually be detected after the training intervention for both groups.

IV. With respect to the stop-signal task, maximal theta power enhancements are detected starting at about 220 ms till 600 ms post stimulus, consistent with those reported in the review of Huster et al. (2013), these power enhancements are very similar before and after training.

V. To inspect training induced changes in fm-theta more closely, pre-post fm-theta difference scores, representing changes in fm-theta power after training compared to the initial power before training, are depicted in Figure 7 (lower panels of A–D) for each EF condition of the four EFs task and groups (NF and pseudo-NF group). As can be seen in Figure 7, fm-theta is decreasing in the pseudo-NF group after the control intervention (an exception is the congruent condition in the Stroop task), whereas the NF group shows amplitude increases. An exception is the stop-signal task, in which the fm-theta activity was decreasing for both groups. Indeed, the training intervention led to significantly different fm-theta changes between the NF and the pseudo-NF group as indexed by the multivariate assessment that revealed a multivariate main effect of GROUP (Wilks’ *λ* = 0.468, *F*_(9,25)_ = 2.774, *p* < 0.05), meaning an increase of 0.0036 dB in the NF group and a decrease of −0.2122 dB in the pseudo-NF group over all conditions and tasks. Univariate analyses for the effects of the group type significantly predicted fm-theta changes in the three-back task, such as significant fm-theta reductions were observed in the zero-back condition (*p* < 0.05) in the pseudo-NF group. A trend (*p* < 0.1) was furthermore observed within the univariate analyses for the prediction of group type concerning fm-theta power changes in the incongruent condition.

## Discussion

Since fm-theta has been proposed as neural “working language” of brain communication for EFs, the current study investigated the effects of an individualized and adaptive eight session NF training to up-regulate fm-theta compared to a pseudo-NF intervention by utilizing a pre/post-test training design, analyzing memory updating, set-shifting, conflict monitoring, and motor inhibition. As a result, it was shown that proper NF training led to facilitated memory updating and mental set shifting, two EFs relying on proactive control, but not to enhanced conflict monitoring and motor inhibition, two EFs that built on reactive control. Furthermore, analyses of neural effects after NF training demonstrated that learning to self-upregulate fm-theta during NF translated to fm-theta changes in tasks engaging EFs after proper training compared to the pseudo-NF intervention. In the following, aspects concerning the subtypes of EFs, the fm-theta NF protocols, and the neural transfer effects are discussed.

With regard to the dual nature of the findings, the differential NF training effects of this fm-theta NF protocol on EFs reflect the current qualitative distinction of cognitive control mechanisms into proactive and reactive control. Indeed, a dual mechanisms framework (DMS; Braver, 2012) has been suggested that works by means of these two distinct operating modes that probably differ concerning their temporal dynamics and relevant neural networks. As such, within the DMS, proactive control is conceptualized as an anticipatory mechanism, actively maintaining task goals that serve as a source of top-down bias, thus supporting facilitated processing of expected events with a high cognitive demand before they actually occur. For instance, in the three-back task, subjects can process upcoming targets by subvocally repeating the sequence they have to keep in WM, and comparing it with their mental representation of the target stimulus. In contrast, reactive control is conceptualized as a reactive bottom-up mechanism that is recruited only when it is required, for instance when interference is detected by a conflict monitoring system or when a stop-signal requires the inhibition of an initiated response as is the case with the Stroop and the stop-signal tasks.

Interestingly, although all EFs, regardless of whether they require proactive or reactive control mechanisms, recruit the superordinate cognitive control network (Niendam et al., 2012), the subtypes seem to rely on different sub-networks. Menon (2011) distinguishes the central executive network, a fronto-parietal system, anchored in the dorsolateral prefrontal cortex (DLPFC) and lateral posterior parietal cortex (PPC), crucially involved in actively maintaining task goals, from the salience network, a cingulate-frontal-opercular system, reacting to detected task events. Similarly, Dosenbach et al. (2008) proposes a dual-network architecture for EFs based on graph analytic methods, differentiating the fronto-parietal from a cingulo-opercular network, implementing proactive and reactive cognitive control, respectively.

From the lack of behavioral effects in the Stroop- and stop-signal tasks, one might be tempted to conclude that fm-theta is not necessarily crucial for the implementation of reactive control. Yet, the current fm-theta protocol of this study might have primarily targeted fm-theta used in the proactive control network and might less effectively address the network implementing reactive control, as fm-theta might primarily affect the detection signals generated in the MCC and less the resolution activity or the compensatory mechanisms processed in other brain regions of the EFs network (Botvinick et al., 2004). Indeed, fm-theta is thought to enable the transmission of information over different cortical brain areas by entraining activity in disparate neural systems (Cavanagh and Frank, 2014). More precisely, the properties of theta oscillations showing high-amplitude-low-frequency modulations denote an ideal neural parameter for neural organization over distal brain regions (Buzsáki and Draguhn, 2004). Theta phase synchrony between the MCC and distal brain regions has been observed in different studies on EFs, such as the study of Cohen and Cavanagh (2011), who reported of single-trial phase synchrony between the MCC and lateral prefrontal brain areas that was modulated by RTs during conflict monitoring. Nigbur et al. (2011) presented enhanced synchrony in the theta range between fronto-medial and lateral frontal electrode sites, interpreted as cooperated work to allocate control during conflict monitoring, as well as between fronto-medial electrode sites with those over the contra-lateral motor area during conflict monitoring, possibly reflecting the renewed need of response selection during conflicts. Just recently, Oehrn et al. demonstrated by means of intra-cranial recordings that fm-theta originated from MCC as conflict detection signal, causally leading to entrained theta in the DLPFC, and finally accomplishing a coupling between DLPFC-gamma power and MCC oscillations for conflict resolution (Oehrn et al., 2014). Ultimately, accounting for the dual nature of EFs provides potentially new avenues for more elaborative feature extraction procedures concerning fm-theta NF protocols.

Apart from the beneficial behavioral effects of proactive control, the current study revealed an overall alteration of fm-theta in EF-tasks after proper NF training as compared to the active control intervention. This finding generally demonstrates that the targeted EF network has been affected by fm-theta NF. However, one would have expected EF-specific fm-theta effects in the proactive tasks to be paralleled by the above described behavioral effects. When looking more specifically at the neural effects, this does not seem to be the case, since results demonstrate that fm-theta effects between the proper NF group and the active control group are detected in the non EF-condition of memory updating, whereas no differences in fm-theta changes were found between both groups with task-switching. Although both the behavioral and the neural findings are interesting in itself, one should keep in mind that only one of the possible neural parameters, namely the fm-theta amplitude, has been assessed. It is conceivable that the fm-theta NF training could have influenced other neural parameters, such as coherence, frequency-coupling, or even myelination, paralleling the behavioral effects in proactive control in a more consistent way. The behavioral effects observed in proactive control could also correspond to structural changes within axonal pathways of relevant networks (e.g., changes in the integrity of white matter or the velocity of conductivity). In fact, glance at the EEG-NF literature indicates that the neural mechanisms underlying NF and putative transfer effects to neurocognition are still not well understood. Little is known about which specific network elements are changed and how the elements are altered due to NF. Behavioral performance increases aren’t necessarily associated with increases in neural activity; rather, reduced neural activity may also result from stimulus and task repetition (see review Grill-Spector et al., 2006). Furthermore, associations between behavioral task proficiency and neural activity after training do not necessarily have to follow a linear function. For example, domain-proficient relative to domain-naive participants may show lower neural activity with simple tasks, but stronger neural processing with higher task difficulty (e.g., Prat and Just, 2011; Dunst et al., 2014). Hence, complex interactions of repetition-related performance changes, training-induced changes in neural responding and behavior, and task difficulty may exist.

Those functional and structural studies, on neural mechanism mediating behavioral improvements, undertaken so far either focus: (a) on immediate and long-lasting functional EEG effects, (b) on immediate fMRI effects and network activity; and finally (c) on long-lasting structural effects found in white matter pathways, usually examined via diffusion tensor imaging (DTI). For instance, Egner and Gruzelier (2001, 2004) were among the first to study neural changes induced by NF by using a protocol to increase activity in the 12–18 Hz frequency range. In their study EEG training effects were accompanied by improved attention. More importantly, after NF an increase of the oddball P300 was detected and interpreted as indicator for improved neural integration of relevant environmental stimuli. In order to investigate immediate neural effects after NF, Ros et al. (2010) examined possible changes of motor evoked potentials (MEP) by means of transcranial magnetic stimulation (TMS) to assess variations in the strength of neurotransmission from the motor cortex to the muscle. By using two types of TMS protocols (single vs. paired pulses), they could dissociate increases in cortico-spinal excitability (CSE), short-interval intra-cortical inhibition (SICI), and intra-cortical facilitation (ICF). This way, Ros et al. (2010) demonstrated that down-regulation of alpha oscillations, considered as a marker of cortical activation, indeed led to increased CSE and decreases of SICI. Similar increases of SICI were recently reported after completion of a 20 session theta/beta training (Studer et al., 2014). Here as well, a decreased P300 observed in the Attention Network Test (ANT) was considered to indicate increased efficiency of stimulus processing. Later on, Ros et al. (2013) conducted an independent component analysis (ICA) of brain activity recorded via fMRI during an oddball task to identify functionally coupled brain regions and to assess putative connectivity changes directly after a single session NF intervention. Immediately after alpha down-regulation, increased activation of brain regions belonging to the salience network were detected, namely within the MCC, the bilateral insular, thalamic, basal ganglia, cerebellar and ponto-mesencephalic regions. Structural changes after NF were assessed by DTI by Ghaziri et al. (2013) after a 3 months beta up-regulation training that resulted in enhanced sustained attention as evidenced in behavioral measures. Interestingly, microstructural changes were observed in three white matter pathways that are part of the sustained attention network, namely the cingulum bundle (a tract connecting the MCC with the DLPFC and the PPC), the anterior corona radiata (connecting the frontal cortex with the brainstem), and the splenium of the corpus callosum (a tract supporting inter-hemispheric processing), which seem not only to speak for enhanced neural transmission, but for increased myelination and faster conduction velocity (CV) underlying improved performance triggered by NF.

Regarding the relation between NF and myelination, participants learn to change oscillatory activity by recruiting and synchronizing the activity of neurons in a specific brain region, which again may affect myelination that preferable takes place at electrically active axons (e.g., Demerens et al., 1996; Ishibashi et al., 2006; Fields, 2010; Wake et al., 2011). Myelin plasticity has been furthermore suggested as an experience and learning dependent mechanisms (Wang and Young, 2014), which is thought to be relevant throughout adulthood (Miller et al., 2012a). Overall, myelin plasticity seems to crucially reflect an important lifelong process of relevance for oscillatory activity and cross-regional coupling (e.g., Pajevic et al., 2014; O’Rourke et al., 2014), and thus may also represent important mechanisms underlying NF-induced neural modulations.

Altogether, self-regulation of endogenous fm-theta increases capacities of EFs and represent a feasible neuroscientific training approach. Theta protocols may also be adapted according to the specific needs of the participants, with a focus on enhancing general EFs or proactive and reactive subtypes, in neurological and psychiatric disorders. Given that age-related changes of fm-theta seem to be related to EFs declines in age (Kardos et al., 2014), fm-theta NF protocols may also serve as an ideal tool to decelerate aging effects.

## Conflict of interest statement

The authors declare that the research was conducted in the absence of any commercial or financial relationships that could be construed as a potential conflict of interest.

## Footnotes

^1^Version 14.8, www.neurobs.com

^2^freely available from http://www.sccn.ucsd.edu/eeglab/
